# Advancing proton minibeam radiation therapy: magnetically focussed proton minibeams at a clinical centre

**DOI:** 10.1038/s41598-020-58052-0

**Published:** 2020-01-28

**Authors:** Tim Schneider, Ludovic De Marzi, Annalisa Patriarca, Yolanda Prezado

**Affiliations:** 1 Université Paris-Saclay, CNRS/IN2P3, IJCLab, 91405 Orsay, France; 2 Université de Paris, IJCLab, 91405 Orsay, France; 3 Institut Curie, University Paris Saclay, Radiation Oncology Department, Centre de protonthérapie d’Orsay, Orsay, France; 4 Institut Curie, University Paris Saclay, PSL Research University, Inserm U 1021-CNRS UMR 3347, Orsay, France

**Keywords:** Radiotherapy, Physics

## Abstract

Proton minibeam radiation therapy (pMBRT) is a novel therapeutic strategy that has proven to significantly increase dose tolerances and sparing of normal tissue. It uses very narrow proton beams (diameter ≤1 mm), roughly one order of magnitude smaller than state-of-the-art pencil beams. The current implementation of pMBRT with mechanical collimators is suboptimal as it is inflexible, decreases efficiency and produces additional secondary neutrons. As a potential solution, we explore in this article minibeam generation through magnetic focussing and investigate possibilities for the integration of such a technique at existing clinical centres. For this, a model of the pencil beam scanning (PBS) nozzle and beam at the Orsay Proton Therapy Centre was established and Monte Carlo simulations were performed to determine its focussing capabilities. Moreover, various modifications of the nozzle geometry were considered. It was found that the PBS nozzle in its current state is not suitable for magnetic minibeam generation. Instead, a new, optimised nozzle design has been proposed and conditions necessary for minibeam generation were benchmarked. In addition, dose simulations in a water phantom were performed which showed improved dose distributions compared to those obtained with mechanical collimators.

## Introduction

Proton minibeam radiation therapy (pMBRT)^1^ is a novel therapeutic strategy based on a distinct spatial modulation of the dose. In contrast to standard proton therapy, the irradiation is carried out with minibeams (very narrow beams with a diameter $\le 1$ mm) separated by gaps of 2 to 4 mm. This results in a lateral dose profile consisting of a succession of areas of high dose (peaks) and areas of low dose (valleys). The ratio between the peak and valley doses (peak-to-valley dose ratio, PVDR) is a biologically relevant parameter: high PVDR with low valley doses favours normal tissue sparing^2^.

Several animal experiments have demonstrated that pMBRT reduces normal tissue damage compared to conventional seamless irradiations^3,4^. In addition, an equivalent or superior tumour control has been observed in glioma-bearing rats^5,6^. These results suggest the participation of distinct radiobiological phenomena (e.g. microscopic prompt tissue-repair effect^7^) which, however, are not yet completely understood.

Recently, pMBRT was implemented at the Orsay Proton Therapy Centre (ICPO) using a multislit collimator attached at the end of the nozzle. This method has been tested both in passive scattering^8,9^ and pencil beam scanning mode^10^. While such a mechanical collimation presents a straightforward way to implement pMBRT at an existing facility, it comes at the cost of a reduced dose rate and overall efficiency. Furthermore, the collimator becomes an additional source of secondary neutrons, which contribute, however, only little to the patient dose^9^. Lastly, this technique is rather inflexible as it may be necessary to fabricate a new collimator for different patients or patient groups.

An approach to overcome these issues could be the generation of proton minibeams through magnetic focussing. Similar to current pencil beam scanning (PBS) treatments, magnetically focussed and scanned minibeams may offer an efficient and versatile tool to maximise tissue sparing, reduce neutron production and pave the way for 3D intensity-modulated treatment planning in pMBRT. The aim of this work was to evaluate how such a technique could be integrated at a clinical centre. For this, a model of the PBS nozzle at ICPO as well as a new, optimised nozzle design were created and investigated with respect to their focussing limits. While other groups have recently explored the use of narrow proton beams (diameter $\ge 3$ mm) to improve conventional PBS treatment^11^ and proton beam radiosurgery^12^, this work is the first one to evaluate magnetically focussed proton minibeams for pMBRT at a clinical centre.

## Material and Methods

This work comprises two principal parts. First, a model of the PBS nozzle at ICPO (IBA Proteus® PLUS universal nozzle) was created and its focussing limits were investigated through Monte Carlo simulations. Additionally, modifications of the nozzle geometry were considered and the resulting effects on the focussing limit were analysed. The second part deals with a new, improved nozzle design capable of delivering clinical proton minibeams. Different parametrisations of the beam at the nozzle entrance were considered to benchmark necessary conditions for minibeam generation and simulations of the dose distribution in a water phantom were performed.

### Monte Carlo simulation details

All presented studies are Monte Carlo simulations of proton beams propagating through a clinical nozzle. The simulations were performed with TOPAS^13,14^ version 3.2 (dose simulations, section 2.3) and TOPAS version 3.1.p3 (all other cases). The physics list was built using the *Geant4_Modular* option and the recommended modules for proton therapy^15–17^. The range cut (all particles) was 10 $\mu$m for the dose simulations and 50 $\mu$m in all other cases, i.e. nozzle and beam modelling as well as beam size minimisation. An energy spread of 0.001% was assumed for the minimisation studies of the PBS nozzle at ICPO. Such a monochromatic beam represents the best case scenario for magnetic focussing and thus prevents an overestimation of the minimum beam size. For the studies of the optimised nozzle design, the energy spread was 1% which is a typical value for facilities using cyclotrons^18^.

### Focussing capabilities of the PBS nozzle at ICPO

As a starting point, the focussing capabilities of an existing clinical nozzle were assessed. For this, a model of the PBS nozzle at ICPO and the beam at its entrance was created. Various model parameters were manipulated to determine the focussing limits.

#### Nozzle and beam model

**Figure 1:**
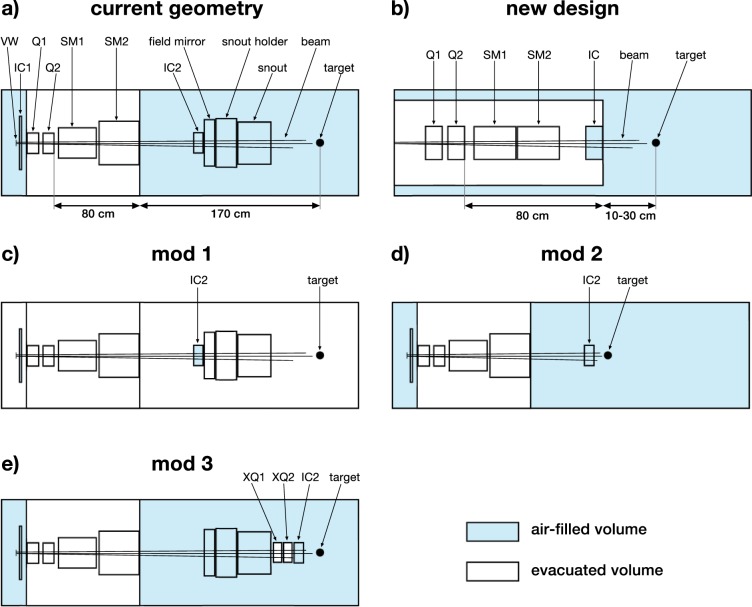
Schematics of the different nozzle geometries presented in this article. Abbreviations stand for: VW - vacuum window, VT - vacuum tank, IC - ionisation chamber, (X)Q - (extra) quadrupole, SM - scanning magnet, SH - snout holder. (**a**) Model of the PBS nozzle at ICPO. (**b**) New, optimised nozzle design. (**c**) Modification of ICPO nozzle with fully evacuated beam path (except interior of ICs). (**d**) Modification of ICPO nozzle without snout and field mirror and shortened path length to target. (**e**) Modification of ICPO nozzle with extra pair of quadrupole magnets and shifted IC2.

The geometric model of the nozzle was created in TOPAS according to plans provided by the manufacturer (see also De Marzi *et al*.^18^). A schematic is shown in Fig. 1a. To obtain a realistic beam focussing model, the magnetic quadrupole fields in Q1 and Q2 were simulated using the *QuadrupoleMagnet* feature in TOPAS. The according field strengths were recorded during experiments.

The accuracy of the *QuadrupoleMagnet* model was validated against the professional software Lorentz 3D-M^19–21^. For this, a pair of quadrupoles and a drift space of 2 m were simulated with either software and the beam size was assessed at multiple positions. The beam sizes agreed on average within 1.3% with a maximum deviation of 5.9% which was considered sufficient for our purposes. More details can be found in the Supplementary Material.

The beam source was modelled at the nozzle entrance and positioned at the vacuum window. An *Emittance* type source was used which defines the beam through six parameters: ${\sigma }_{x},\,{\sigma }_{y},\,{x}^{^{\prime} },\,{y}^{^{\prime} },\,{r}_{x{x}^{^{\prime} }}$ and ${r}_{y{y}^{^{\prime} }}$. The parameters ${\sigma }_{x},\,{\sigma }_{y}$ give a measure of the horizontal and vertical beam size while ${x}^{^{\prime} },\,{y}^{^{\prime} }$, representing the *beam divergence*, describe the horizontal and vertical angular spread of the beam. The factors ${r}_{x{x}^{^{\prime} }}$ and ${r}_{y{y}^{^{\prime} }}$ denote the correlation coefficients between ${\sigma }_{x}$ and ${x}^{^{\prime} }$ and ${\sigma }_{y}$ and ${y}^{^{\prime} }$. A bi-Gaussian profile was assumed for both the particle positions and momenta which means that ${\sigma }_{x},\,{\sigma }_{y}$ and ${x}^{^{\prime} },\,{y}^{^{\prime} }$ correspond to the standard deviations of the respective Gaussian distributions.

**Figure 2:**
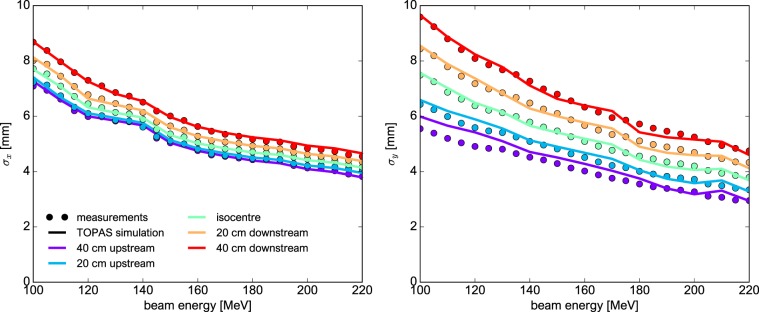
Comparison of the horizontal (left) and vertical (right) beam sizes obtained in TOPAS (solid lines) with measurements (circles) at five different positions around the isocentre and for beam energies from 100 to 220 MeV. The sizes refer to the standard deviation of the respective Gaussian distribution (cf. text section 2.2.1). Uncertainty bars are smaller than markers/line width.

Values for ${\sigma }_{x}$ and ${\sigma }_{y}$ were obtained by measuring the beam size in IC1 (see Fig. 1a). The remaining beam parameters were determined using a best-fit approach comparing measured beam sizes to the beam sizes simulated with different source parameterisations. The comparison was done at five positions around the isocentre ($-40$ cm, $-20$ cm, isocentre, +20 cm and +40 cm) and for thirteen beam energies from 100 to 220 MeV (see Fig. 2). Measuring the beam in this range corresponds to a clinically relevant situation (patient size) and provides a more accurate estimation of the beam divergence at the isocentre.

#### Beam size minimisation with current nozzle geometry

The model of the PBS nozzle was used to determine the minimum beam size that can be achieved with the current geometry. Beam energies of 100, 150 and 200 MeV were considered. For this first step, only the target position and the configuration of the magnetic quadrupoles were varied. The considered target positions were $-40$, $-20$, 0, +20 and +40 cm relative to the original isocentre and the quadrupole configurations were specified by the magnetic field strength and the orientation of the focussing plane. Quadrupoles always focus in one plane and defocus in the orthogonal plane, thus two arrangements were distinguished: Q1 focussing horizontally with Q2 focussing vertically, and vice versa. The field strength was defined by the field at the pole tips and was incremented in steps of 0.04 T from 0 to 2 T, resulting in 51 different field strengths for each quadrupole.

The minimum beam size was then determined by comparing the sizes simulated with each of the $51\times 51\times 2$ quadrupole configurations. At each of the five positions, phase space actors were used to record the horizontal and vertical beam profiles which were fitted with a Gaussian distribution. The full width at half maximum (FWHM) of the Gaussian was used as a measure for the beam size. It relates to the aforementioned standard deviation $\sigma$ of the Gaussian as ${\rm{FWHM}}\approx 2.355\,\sigma$. For simplification, we will distinguish the horizontal FWHM (hFWHM) and the vertical FWHM (vFWHM).

A minibeam must thus satisfy that both the hFWHM and vFWHM are $\le 1$ mm which implies a simultaneous minimisation of the beam size in horizontal and vertical direction in a more or less symmetric fashion. This minimisation requires a scalar quantity that takes into account the hFWHM and vFWHM as well as a factor ensuring that both FWHM are small. Such a quantity is defined in equation (1).

1 $$\Omega :=\left({\rm{hFWHM}}\cdot {\rm{vFWHM}}\right)\cdot \left(\frac{{\rm{hFWHM}}}{{\rm{vFWHM}}}+\frac{{\rm{vFWHM}}}{{\rm{hFWHM}}}\right)={{\rm{hFWHM}}}^{2}+{{\rm{vFWHM}}}^{2}.$$

**Figure 3:**
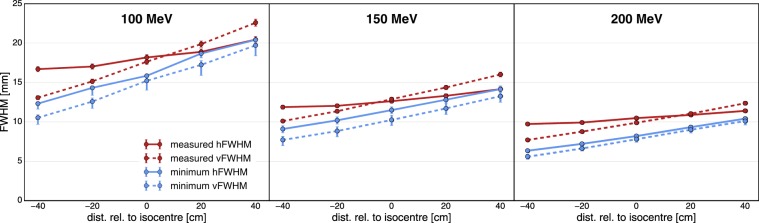
Minimum beam sizes achievable with the current PBS nozzle at ICPO for beam energies of 100, 150 and 200 MeV. The x-axes state the target position relative to the original isocentre. Red: reference beam sizes measured at ICPO. Blue: minimised beam sizes. The hFWHM and vFWHM are given by solid and dashed lines, respectively.

The minimum beam size is then given by the hFWHM and vFWHM of the quadrupole configuration yielding the minimum $\Omega$. A graphic representation of this can be found in the supplementary material. The values of $\Omega$ have no physically relevant meaning and are therefore not stated. Fig. 3 shows the results of this minimisation process.

The minimum beam size is affected by uncertainties of the beam model. An estimation for this was obtained by simulating several variations of the beam source parameters. Furthermore, the true minimum may be missed due to the discrete magnetic field strengths used during the minimisation process. This uncertainty was approximated by the local variation of the beam size around the minimum configuration. It can only overestimate the true minimum and thus contributes asymmetrically to the total uncertainty. Lastly, the fitting process introduces a small uncertainty for which an estimate was provided directly by the algorithm. The uncertainty bars shown in Fig. 3 were computed as the root of the squares of these three contributions.

#### Beam size minimisation with modified geometries

After considering the PBS nozzle in its current state, several (theoretical) modifications of the nozzle geometry were simulated to evaluate different approaches for further beam size reduction. Schematics of these modifications are shown in Fig. 1c–e.

The first approach (Fig. 1c) was targeted at beam widening due to scattering in air. All volumes except the ionisation chambers (IC1, IC2) were evacuated. The air in the ionisation chambers acts as the gas that is ionised and cannot be removed without changing the functional design. In a second modification (Fig. 1d), the field mirror, snout holder and snout, which serve no purpose in this case, were removed so that the target could be moved closer to IC2. This reduced the total length of the beam path and also shortened the distance where the beam travels in air. The third modification (Fig. 1e) extended the original geometry by adding a quadrupole pair downstream of the snout, thus moving the last focussing element much closer to the target. Moreover, IC2 was moved downstream of the added quadrupoles to account for the practical constraint that elements manipulating the beam must be followed by a beam monitoring unit.

**Figure 4:**
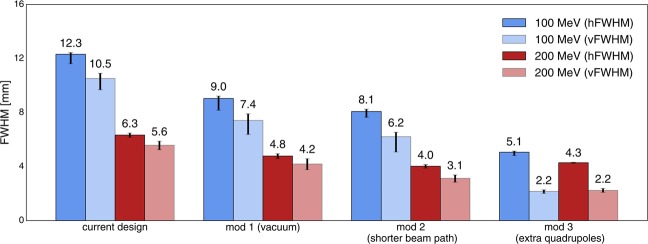
Comparison of the minimised beam sizes for the current nozzle geometry and its modifications. From left to right, the cases correspond to panels (a,c–e) in Fig. 1.

The considered beam energies were 100 and 200 MeV. The minimum beam size was again assessed by systematically changing the quadrupole configurations, as described in the previous section. For the quadrupoles XQ1, XQ2 (Fig. 1e), values of the magnetic field at the pole tips up to 4 T were considered. Figure 4 shows the minimised beam sizes for each modification. The uncertainties were estimated in the aforementioned way.

### Optimised design

A new, more compact nozzle design was proposed based on the insights obtained with the model of the current nozzle and its modifications. A schematic of the geometry is shown in Fig. 1b. The design mostly uses the same elements as the PBS nozzle but features a much shorter focal length and air gap. Two different air gap sizes, 10 and 30 cm, were considered as well as two different beam energies, 100 and 200 MeV.

#### Beam parameters at the nozzle entrance

Apart from the nozzle geometry, the final beam size also depends on the initial beam parameters. The central parameter in this context is the beam emittance which represents a measure of the spread of the individual particle positions and momenta. As a consequence of Liouville’s theorem, the emittance cannot be changed by conservative forces^22^ which implies that minibeams suitable for pMBRT, i.e. with a low divergence, can only be generated if the beam exhibits an adequate beam emittance to begin with.

In order to benchmark the according parameters, various parameterisations of the beam at the nozzle entrance were simulated for each air gap length and beam energy. They were constructed by varying the six beam source parameters used by TOPAS’ *Emittance* source. Equation (2) expresses the beam emittance in terms of these parameters.

2 $${\varepsilon }_{x}\propto {\sigma }_{x}\cdot {x}^{^{\prime} }\cdot \sqrt{1-{r}_{x{x}^{^{\prime} }}^{2}}$$

It shows that the emittance becomes small when ${\sigma }_{x}$ or ${x}^{^{\prime} }$ are small or $| {r}_{x{x}^{^{\prime} }}|  \sim 1$. It should be noted that an emittance is defined for each of the transversal planes and the same also holds true when $x$ is replaced with $y$.

**Figure 5:**
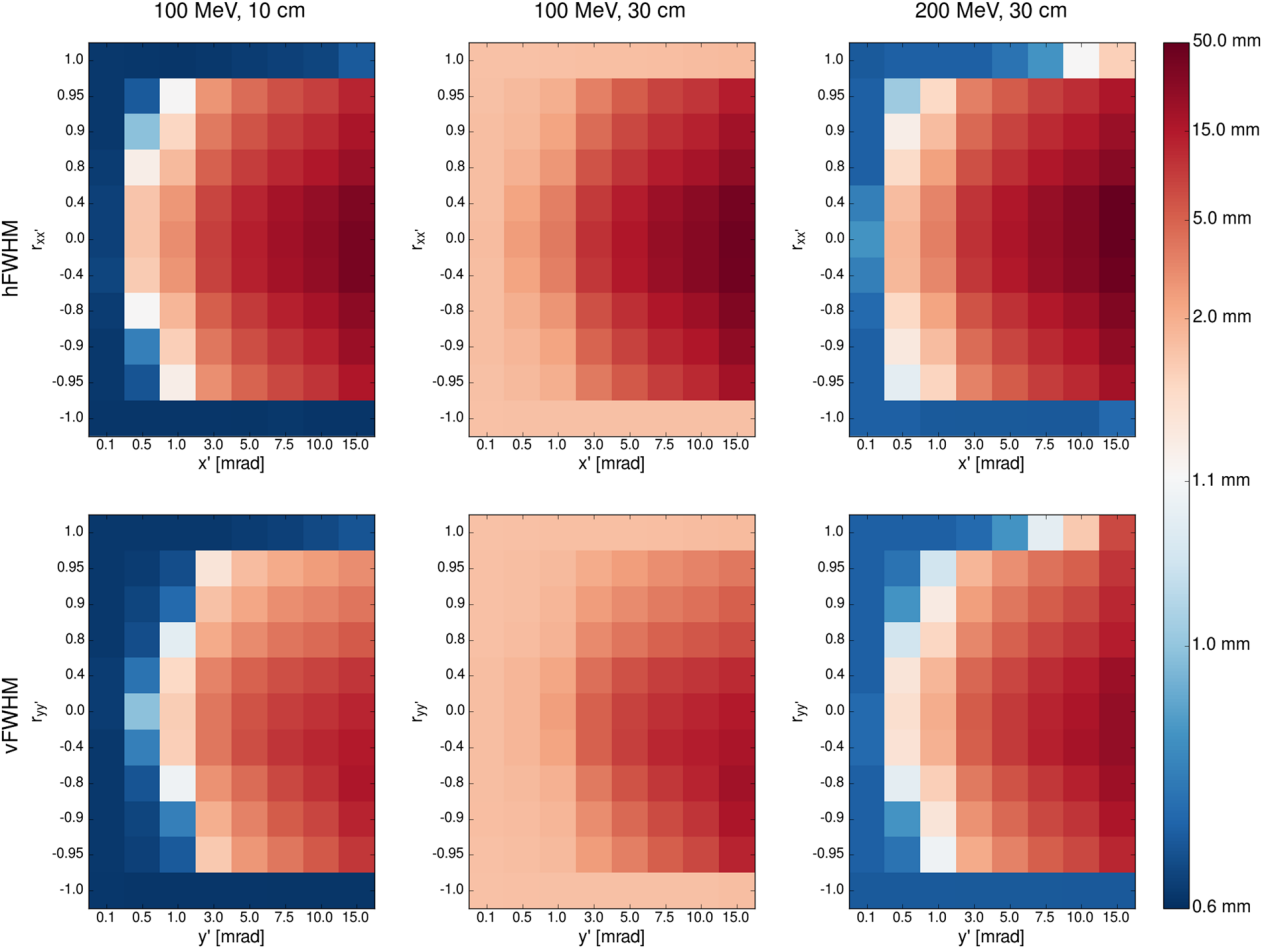
Minimum beam size achievable with the optimised nozzle design for different parameters of the beam at the nozzle entrance. A blue colour represents a beam parametrisation suitable for minibeam generation. Top row: minimal hFWHM as function of ${x}^{^{\prime} }$ and ${r}_{x{x}^{^{\prime} }}$. Bottom row: minimal vFWHM as function of ${y}^{^{\prime} }$ and ${r}_{y{y}^{^{\prime} }}$. Note that possible values of the correlation coefficient range from $-1$ to +1 and that the scales of the x- and y-axes are not linear.

For the simulations, nine initial beam sizes $({\sigma }_{x},{\sigma }_{y})$ were distinguished where $3\le {\sigma }_{x},\,{\sigma }_{y}\le 15$ mm as well as eight values between 0.1 and 15 mrad for ${x}^{^{\prime} }$ and ${y}^{^{\prime} }$ each and eleven values between $-1$ and +1 for ${r}_{x{x}^{^{\prime} }}$ and ${r}_{y{y}^{^{\prime} }}$ each, yielding a total of almost 70,000 considered parametrisations. The beam source was always at the entrance of the vacuum tank (left border in Fig. 1b) and the same process described in section 2.2.2 (field strengths $\le 2$ T) was used to determine the minimum beam size. Examples of how the minimum beam size changes for different initial parametrisations are displayed in Fig. 5 while the absolute minima are listed in Table 1. The uncertainty on the beam size in Table 1 includes uncertainties introduced by the fitting algorithm and due to the discrete values of the magnetic fields used for the minimisation process.

#### Dose distributions

**Figure 6:**
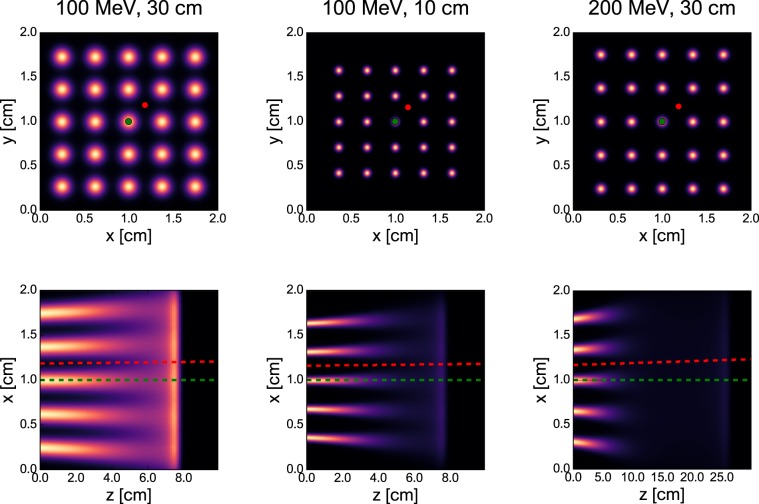
Dose distributions of minibeam grids in water phantom simulated for the three configurations listed in Table 1. Top row: transversal distribution at the phantom entrance. Bottom row: longitudinal distribution through the centre of the phantom ($y=1$ cm). Green dots and dashed lines: peak regions considered in Fig. 7. Red dots and dashed lines: valley regions considered in Fig. 7.

Three of the considered configurations were selected for simulations of the dose distribution in a water phantom ($2\ {\rm{cm}}\times 2\ {\rm{cm}}\times 10\ {\rm{cm}}$ for 100-MeV beams and $2\ {\rm{cm}}\times 2\ {\rm{cm}}\times 30\ {\rm{cm}}$ for 200-MeV beams). The irradiation field consisted of $5\times 5$ minibeams arranged in a square grid with a spacing leading to lateral homogenisation at the Bragg peak depth for the 100-MeV beams (lateral flatness^23^ 5-6%). The center-to-center distances were 2.9 and 3.7 mm for air gaps of 10 cm and 30 cm, respectively, leading to field sizes^23^ of 17.4 and 19.8 mm. A similar spacing was chosen for the 200-MeV case. Dose maps visualising these grids are shown in Fig. 6. The scanning dipoles (SM1 and SM2), simulated using TOPAS’ *DipoleMagnet* feature, were used to deflect the minibeams to their respective positions. As in previous studies^1,24^, the total area covered was approximately $2\times 2\ {{\rm{cm}}}^{2}$.

The dose was recorded with the *DoseToWater* scorer of the TOPAS framework at a voxel size of $0.05\ {\rm{mm}}\times 0.05\ {\rm{mm}}\times 1\ {\rm{mm}}$. For each voxel, the dose uncertainty was calculated by considering the standard deviation of 50 simulations. The global relative uncertainty was then computed as the root mean square of the voxel uncertainties over all voxels with at least half the maximum dose. It was less than 0.83% in all cases.

Finally, the dose distributions were analysed based on the hFWHM of a single minibeam, the PVDR and the depth dose profiles along a peak and a valley region. The hFWHM was assessed for the dose distribution of only the central minibeam without the contribution of the 24 surrounding minibeams. Uncertainties of the FWHM are taken from the fitting algorithm while uncertainty bars for the PVDR were assessed by propagating the dose uncertainties of the corresponding voxels.

## Results and Discussion

### Focussing capabilities of the PBS nozzle at ICPO

This section focusses mainly on the results of the beam size minimisation simulations. More detailed information about the validation of the *QuadrupoleMagnet* feature and the modelling of the beam at ICPO can be found in the Supplementary Material.

#### Beam model

All parameters of the final beam model are functions of the beam energy (see also Supplementary Material). The parameters ${\sigma }_{x}$ and ${\sigma }_{y}$ were inferred from measurements and lie between 3.2 and 13 mm. The other beam source parameters, determined through a best-fit process, are $0.3\le {x}^{^{\prime} },{y}^{^{\prime} }\le 2.25$ mrad and $-1.0\le {r}_{x{x}^{^{\prime} }},{r}_{y{y}^{^{\prime} }}\le 1.0$. Figure 2 compares the measured beam sizes to the ones obtained with the final beam model. Overall, a good agreement is observed with a mean deviation of 0.9% and 3.2% for ${\sigma }_{x}$ and ${\sigma }_{y}$, respectively.

#### Current nozzle geometry

The focussing capabilities of the PBS nozzle at ICPO were assessed at five different positions by simulating $51\times 51\times 2$ quadrupole configurations as explained in section 2.2.2. The exact values of the quadrupole fields are specific to the geometry and not stated here. Some examples can nevertheless be found in the Supplementary Material.

Figure 3shows the resulting minimum beam sizes along with measurements of the current beam sizes at ICPO. The beam sizes are given as a function of the target position and for beam energies of 100, 150 and 200 MeV. Note that each minimised beam size corresponds to a distinct quadrupole configuration.

Although a considerable reduction of the beam size was achieved (20–25% smaller than the measured values), submillimetric beam sizes could not be obtained. The smallest FWHM, which was attained 40 cm upstream of the isocentre at a beam energy of 200 MeV, still amounts to 5.6 mm. Thus, it can be concluded that the generation of purely magnetically focussed minibeams is most likely not possible with the current PBS nozzle. Very different minima are obtained at the various positions. From $-40$ to +40 cm, the minimum beam size increases by almost 100% at all considered beam energies. This highlights the importance of the nozzle-to-target distance.

#### Modified nozzle geometries

The PBS nozzle in its current state will not be able to provide sufficient focussing of the beam. To identify the limiting factors, various modifications of the nozzle geometry were investigated. Figure 4 compares the minimum beam sizes obtained with these modifications to the minima of the current geometry.

The evacuation of air from the current geometry (mod 1) leads to a remarkable reduction of the beam size of 25–30%, illustrating the importance of scattering in air. A reduction by a further 10–25% is obtained with a shorter beam path (mod 2). This result can still be improved with the additional quadrupole pair (mod 3) leading to a FWHM of less than 2 mm at 100 MeV.

A common feature shared by modifications 2 and 3 is that the distance between the last focussing element (last quadrupole) and the target is much shorter than in the current configuration at ICPO. Consequently, also the path in air is reduced which means fewer scattering events and thus a lower beam broadening. More importantly however, the focal length gets shortened which, in turn, makes it easier to focus beams with a high emittance.

This behaviour is because a higher emittance indicates a larger spread of the particle positions and momenta. The spread of the particle momenta is especially important for long focal lengths which correspond to small deflection angles. Conversely, short focal lengths which require large deflection angles are less sensitive to these variations. Hence, the smallest beam sizes were obtained with mod 3 where the focal length was of the order of 30 cm. A drawback of very short focal lengths, however, is that, due to the large deflection angles, the beam will diverge rapidly downstream of the focal point. In practice, this becomes important as it is desirable to maintain a submillimetric beam size over a distance of several centimetres. At any rate, these results point out the need for a new, optimised design which is discussed in the next section.

### Optimised design

The evaluation of the modified geometries showed that the minimum beam size can be efficiently reduced by shortening the focal length and the length of the air gap. Furthermore, it was argued that a too short focal length will result in very divergent beams. Accordingly, a new, optimised nozzle design was investigated that incorporates a moderate focal length distance from the exit plane of Q2 to the target between 90 and 110 cm as well as a short air gap of 10 to 30 cm (cf. Fig. 1b).

#### Beam parameters at the nozzle entrance

In order to benchmark initial beam parameters suitable for minibeam generation, various different beam source parametrisations were simulated as explained in section 2.2.1. For each parametrisation, the minimum beam size was determined, as before, by variation of the quadrupole fields. It was found that the focussing capabilities mainly depend on the divergence and correlation coefficient while the initial beam size is virtually irrelevant. Furthermore, the final hFWHM is predominantly affected by ${x}^{^{\prime} }$ and ${r}_{x{x}^{^{\prime} }}$, while the vFWHM depends mainly on ${y}^{^{\prime} }$ and ${r}_{y{y}^{^{\prime} }}$. In other words, the horizontal and vertical plane are not correlated.

Figure 5 illustrates these findings by plotting the hFWHM and vFWHM as functions of $({x}^{^{\prime} },\,{r}_{x{x}^{^{\prime} }})$ and $({y}^{^{\prime} },\,{r}_{y{y}^{^{\prime} }})$, respectively. The colour of the squares represents the magnitude of the minimum FWHM for the corresponding initial parameters. A blue colour indicates a FWHM of $\lesssim 1$ mm and thus marks a parametrisation suited for minibeam generation. Three cases of different beam energies and air gap lengths were considered: 100-MeV beam and 30-cm air gap, 100-MeV beam and 10-cm air gap and 200-MeV beam and 30-cm air gap. The case of a 200-MeV beam and 10-cm air gap has been omitted as minibeams of this energy could already be obtained with a gap of 30 cm. From a practical standpoint, a larger air gap is preferred as the additional space may be needed e.g. for positioning and imaging systems.

In all cases, the smallest beam sizes are obtained if at least one of two conditions is satisfied: the initial divergence is very small (${x}^{^{\prime} },\,{y}^{^{\prime} } \sim 0.1$ mrad) or the correlation coefficient takes on extreme values ($0.95\le | {r}_{x{x}^{^{\prime} }}| ,\,| {r}_{y{y}^{^{\prime} }}| \le 1$). Nonetheless, a submillimetric vFWHM was also observed for the case of the 100-MeV beam and 10-cm air gap for divergences of 0.5 and 1 mrad and a correlation coefficient $\pm 0.9$. These requirements can be considered realisable as correlation coefficients of $\pm 1$ were already used in the beam source model of the current nozzle at ICPO (see supplementary material and De Marzi *et al*.^18^). Moreover, the beam divergence has undergone an improvement in the last years from values around 9 mrad^25^ to $\sim 1$ mrad^18^ and, in some cases, 0.5 mrad (data kindly provided by the team of MedAustron) and it may be expected that this trend continues.

The minimum beam sizes for each of the three cases are listed in Table 1. Minibeams were obtained for the 100-MeV beams and 10-cm air gap (0.66 mm) and 200-MeV beams and 30-cm air gap (0.85 mm). At 100 MeV and with an air gap of 30 cm, the minimum FWHM was millimetric (1.65 mm) which nonetheless represents a significant improvement compared to current beam sizes at ICPO ($$ > 12$$ mm). Each of the minima was in fact obtained with multiple different source parametrisations. It should be noted that only field strengths up to 2 T were considered which are readily achievable with normal-conducting magnets. The listed parametrisations correspond to those used for the dose simulations (section 3.2.2).

**Table 1:** Minimum beam sizes at the target simulated with new nozzle design for the different combinations of beam energy and air gap length. The sizes were obtained with multiple beam source configurations. The right part of the table lists the source parametrisations used for the dose simulations.

Configuration		Minimum beam size at target		Source beam parametrisation					
beam energy [MeV]	air gap [cm]	hFWHM [mm]	vFWHM [mm]	${\sigma }_{x}$ [mm]	${\sigma }_{y}$ [mm]	${x}^{^{\prime} }$ [mrad]	${y}^{^{\prime} }$ [mrad]	${r}_{x{x}^{^{\prime} }}$	${r}_{y{y}^{^{\prime} }}$
100	30	$1.6{7}_{-0.19}^{+0.001}$	$1.6{5}_{-0.40}^{+0.001}$	6.5	10.0	5.0	5.0	$-1$	$-1$
100	10	$0.6{6}_{-0.11}^{+0.001}$	$0.6{8}_{-0.11}^{+0.001}$	4.0	4.0	3.0	3.0	$-1$	$-1$
200	30	$0.8{7}_{-0.14}^{+0.001}$	$0.8{5}_{-0.07}^{+0.001}$	5.0	3.5	1.0	0.5	1	$-1$

#### Dose simulations

Simulations of the dose distribution in a water phantom were performed for the configurations listed in Table 1. Transversal and longitudinal dose distributions are shown in Fig. 6. The transversal distribution shows that the off-centre minibeams propagate at an angle in the water phantom. This is due to the short distance between the scanning magnets and the phantom which requires comparatively large deflection angles. Field sizes with a radius of 8–10 cm can be obtained which is sufficient in many cases including typical brain cancers which are the main target for pMBRT.

**Figure 7:**
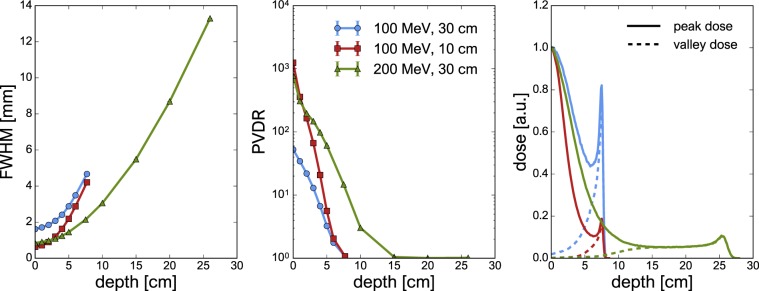
Comparison of the dose distributions simulated for the three configurations listed in Table 1. Left panel: hFWHM of the central minibeam as a function of depth in the water phantom. Central panel: PVDR as a function of depth in the water phantom. Right panel: percentage depth dose curves along peak (solid lines) and valley (dashed lines) regions. Uncertainty bars in the left and central panel are smaller than markers.

 Figure 7 shows the hFWHM of the central minibeam and the PVDR as functions of depth as well as the peak and valley depth dose profiles. The positions where the peak and valley doses were assessed are represented by green and red dots and dashed lines in Fig. 6.

The FWHM of the 200-MeV beam increases much more slowly than those of the 100-MeV beams. This is because beam particles with higher energy have greater forward momentum and are less affected by multiple Coulomb scattering. The two different 100-MeV configurations, despite starting at very different sizes, both reach a FWHM of 4–5 mm at Bragg peak depth. At the same depth ($z=7.5$ cm), the 200-MeV beam has only widened to about 2 mm.

At shallow depths, excellent values of the PVDR $\ge$ 50 are obtained for all cases. Previous studies with mechanical collimators found maximal values of 8–16^8,10^. The highest PVDR ($\sim$1,000) is obtained for a beam energy of 100 MeV with an air gap of 10 cm. It is about 20 times higher than the PVDR for the same energy and a 30-cm air gap. In both cases, the PVDR drops below 10 at a depth of about 3.5–4.5 cm. The PVDR of the 200-MeV configuration reaches initial values around 750 and maintains a PVDR $\gtrsim \ 10$ up to a depth of 8 cm. This is a result of the 200-MeV beams broadening less rapidly which could benefit especially the treatment of deep-seated tumours.

The ratio of the peak dose deposited in the Bragg peak and at the phantom entrance is less than one in all cases. A lower ratio is observed for smaller beam sizes and higher beam energies (0.82 for 100 MeV and 30 cm, 0.19 for 100 MeV and 10 cm and 0.11 for 200 MeV and 30 cm). This is because the fluence in the minibeam centre changes drastically with depth. Very small beams exhibit a high density at shallow depths which decreases as the beam widens. The ratio between the Bragg peak and entrance doses could be improved e.g. using planar minibeams.

## Conclusion

pMBRT presents a new therapeutic strategy that can significantly increase preservation of normal tissue^2–4^ while providing equivalent or superior tumour control^5,6^. The current implementation using mechanical collimators^8–10^ is however suboptimal. In this work, we considered the generation of purely magnetically focussed minibeams and investigated how such a method could be integrated at existing clinical centres.

Starting from the current PBS nozzle at ICPO, which was shown to not be suitable for magnetic minibeam generation, we have proposed a new, optimised nozzle design that uses conventional beamline elements and features a moderate focal length of about 1 m and a shortened air gap of 10–30 cm. An extensive study of different beam source parametrisations demonstrated that either a very small initial divergence ($\lesssim 0.1$ mrad) or an extreme correlation between beam size and beam divergence is necessary for minibeam generation. Under these conditions, the new nozzle design was capable of delivering beam sizes between 0.66 and 1.67 mm FWHM at beam energies of 100 and 200 MeV. The design can thus be considered suitable for pMBRT and could lead to an optimal implementation of pMBRT enabling a more efficient and flexible treatment, accessible to 3D intensity-modulated treatment planning. In addition, dose simulations showed PVDR $\ge \ 50$ for all evaluated cases. This is at least three times higher than the values achieved with mechanical collimators^8,10^ and may further benefit normal tissue sparing.

## Supplementary information

Supplementary Information.
