# Supplementary material for: Advancing proton minibeam radiation therapy: magnetically focussed proton minibeams at a clinical centre
Source: Sci Rep. 2020 Jan 28;10:1384. doi: 10.1038/s41598-020-58052-0 (PMC6987213; doi:10.1038/s41598-020-58052-0)
Supplement: Supplementary file 1 — Supplementary Information. [file 41598_2020_58052_MOESM1_ESM.pdf]

Advancing proton minibeam radiation therapy: magnetically focussed proton minibeams at a clinical centre

Tim Schneider[1,2,*], **Ludovic De Marzi**[3,4], **Annalisa Patriarca**[3], **and Yolanda Prezado**[1,2]

[1]Imagerie et Modélisation en Neurobiologie et Cancérologie (IMNC), CNRS, Univ Paris Sud, Université Paris-Saclay, 91400 Orsay, France
[2]Université de Paris, IMNC, F-91400 Orsay, France
[3]Institut Curie, University Paris Saclay, Radiation Oncology Department, Centre de protonthérapie d'Orsay, Orsay, France
[4]Institut Curie, University Paris Saclay, PSL Research University, Inserm U 1021-CNRS UMR 3347, Orsay, France
[*]schneider@imnc.in2p3.fr

Supplementary material

Comparison of different quadrupole models

A central part for obtaining a realistic nozzle model was the simulation of the quadrupole magnets. In TOPAS, magnetic quadrupole fields can be included using either the *QuadrupoleMagnet* or the *MappedMagnet* option. The *QuadrupoleMagnet* option simulates an idealised, perfect quadrupole field that is defined through a horizontal and vertical field gradient. In the cases considered here, the quadrupoles were always symmetric, so both gradients were equal. The *MappedMagnet* option allows the inclusion of a full 3D map of the magnetic field which enables the simulation of more complex field configurations. Generally, a higher accuracy can be expected from the field maps because inhomogeneities of the field and fringe field effects can be taken into account. However, the field maps cannot be calculated with TOPAS and have to be created first with another software first.

In order to check and compare the accuracy of both approaches, a study was performed with the *QuadrupoleMagnet* and *MappedMagnet* options in TOPAS and with the professional software Lorentz 3D-M[1–3]. Lorentz can compute three-dimensional magnetic field distributions from given electrical currents (the currents in the coils of the quadrupoles) and simulate the transport of charged particles in these fields. The computed field distributions can be sampled and exported to text files and the field maps for the *MappedMagnet* option were created this way.

An identical geometry consisting of a proton beam propagating in vacuum and traversing a pair of quadrupoles was simulated with the two TOPAS approaches and Lorentz. The dimensions and strength of the quadrupoles as well as the length of the beam path were similar to the corresponding values of the PBS nozzle at ICPO. Multiple beam energies between 100 and 220 MeV were considered. The simulations were compared by assessing the beam size at a number of positions along the beam path. Figure 1 shows the resulting horizontal and vertical beam sizes (σ_x and σ_y, respectively) as functions of the position along the beam path.

A good agreement was found between Lorentz and both TOPAS models. The results obtained with the *MappedMagnet* option agreed with the Lorentz simulation on average within 1.7% (σ_x) and 0.8% (σ_y). The maximum deviations were 8.7% for σ_x and 3.2% for σ_y at beam energies of 100 and 130 MeV, respectively. For the *QuadrupoleMagnet* option, the mean deviations were 1.1% (σ_x) and 1.4% (σ_y) and the maximum deviations 3.6% (σ_x, 220 MeV) and 5.9% (σ_y, 130 MeV). In conclusion, both quadrupole models in TOPAS yielded a similarly good accuracy compared to Lorentz and either method could be used for the studies presented in the main text. Considering that an extra calculation is needed for each new field maps, the more straightforward *QuadrupoleMagnet* option was finally chosen.

Definition of the beam source for the model of the PBS nozzle at ICPO

Table 1 lists the parameters of the final beam source model for various beam energies between 100 and 220 MeV. The values for σ_x and σ_y were obtained through measurements with the ionisation chamber at the nozzle entrance (IC1). The remaining parameters were estimated via a best-fit approach by simulating many different beam sources with varying values for x', y' and $r_{xx'}, r_{yy'}$.

In order to determine how well a source parametrisation fits the beam at ICPO, the simulated beam size was compared to measurements at five different positions around the isocentre (-40 cm, -20 cm, 0 cm = isocentre, +20 cm and +40 cm) and the

mean squared error of the five values was computed. The resulting errors are shown in Figure 2. A red colour represents a smaller error (better fit) and the circles indicate the best fitting parameters for each beam energy. Horizontal and vertical beam parameters, $(\sigma_x, x', r_{xx'})$ and $(\sigma_y, y', r_{yy'})$, were considered separately because the two transversal planes are not correlated. The final beam model listed in Table 1 was defined as a smooth interpolation between the best-fit parameters (circles in Figure 2).

Example of the beam size minimisation procedure

The minimisation of the beam size was done in an iterative process simulating the same initial beam with many different configurations of the pair of quadrupole magnets. These configurations differed in the field strength and orientation of the focussing plane of the quadrupoles. Concretely, the field at the pole tips was varied in steps of 0.04 T between 0 and 2 T (4 T for some cases, see main text) and two orientations where the beam is focussed horizontally and vertically, respectively, were considered for each quadrupole. A single quadrupole always focusses in only one direction while defocussing in the orthogonal direction. Nonetheless, a pair of quadrupoles can focus simultaneously in all directions if the focussing plane of the first quadrupole is orthogonal to that of the second one. This implies that a total of $51 \times 51 \times 2$ configurations could be simulated for each beam minimisation. The optimisation procedure had to involve complete Monte Carlo simulations in order to properly account for beam scattering and its contribution to the final beam size.

As stated in the main text, the minimum beam size was found by minimising $\Omega = \text{hFWHM}^2 + \text{vFWHM}^2$ over all $51 \times 51 \times 2$ quadrupole configurations. Figure 3 shows the values of the hFWHM, vFWHM and Ω at the isocentre for the current geometry of the PBS nozzle as functions of the field at the pole tips of the quadrupoles Q1 and Q2 (labelled as B_1 and B_2, respectively). The figure displays the case where Q1 focusses horizontally and Q2 focusses vertically. Lighter colours signify smaller values and circles mark the configurations yielding the minima of either quantity.

It becomes apparent that a small value of Ω is obtained only for a few combinations of rather low field strengths ($0.3\text{ T} \leq B_1, B_2 \leq 0.7\text{ T}$). On the other hand, a small hFWHM can be obtained for any B_2 and a small vFWHM is possible for any B_1. For this particular case, the minimum hFWHM is reached for very small fields ($B_1, B_2 \leq 0.12$ T) while the minimum vFWHM requires comparatively high field strengths ($B_1 = 1.28$ T, $B_2 = 1.88$ T). In practice, these values depend on the exact geometry of the quadrupoles which is why they were omitted in the main text.

References

1. https://www.integratedsoft.com/products/lorentz.aspx.
2. Hahto, S. K. & Bilbrough, D. G. Modeling of a high current h- lebt with the lorentz-em 3d ion optics code. *AIP Conf. Proc.* **925**, 318 (2007).
3. Saminathan, S., Beijers, J., Mulder, J., Mironov, V. & Brandenburg, S. Dipole magnet optimization for high efficient low energy beam transport. *Proc. ECRIS2010* 197–199 (2010).

Figures and tables

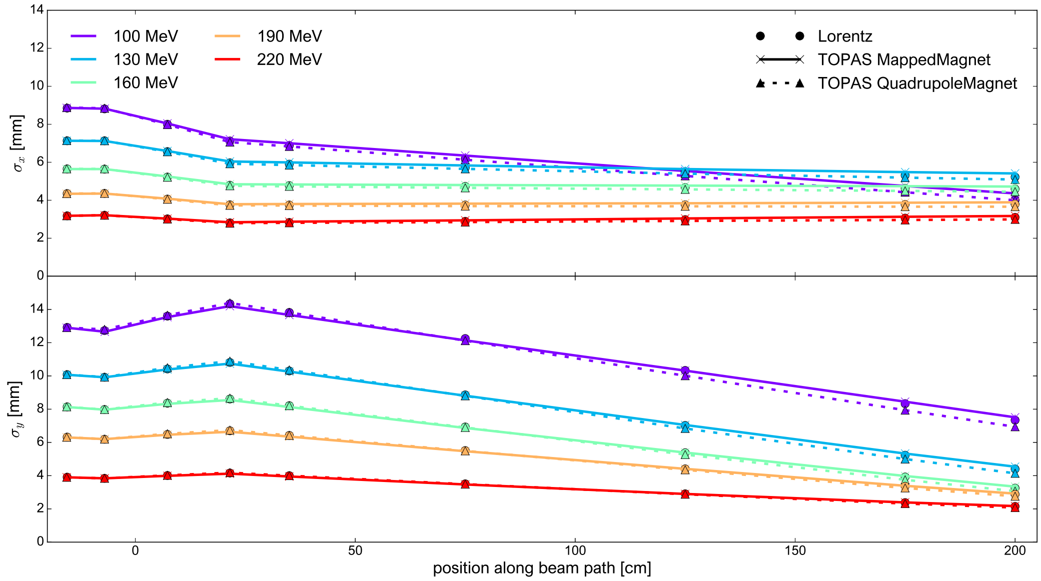

Figure 1. Comparison of the horizontal and vertical beam sizes (σ_x and σ_y) at various positions along the beam path for simulations with Lorentz 3D-M (circles) and TOPAS using the *QuadrupoleMagnet* (triangles and dashed lines) and *MappedMagnet* options (crosses and solid lines).

Beam energy [MeV]	σ_x [mm]	σ_y [mm]	x' [mrad]	y' [mrad]	$r_{xx'}$	$r_{yy'}$
100	8.9	13.0	0.50	2.25	-1.0	-0.9
110	8.3	11.9	0.50	2.25	-0.8	-0.9
120	7.7	11.0	0.50	2.25	-0.6	-0.9
130	7.2	10.1	0.50	2.25	-0.2	-0.9
140	6.6	9.4	0.40	2.00	0.0	-0.9
150	6.1	8.8	0.50	2.00	-0.2	-0.9
160	5.7	8.1	0.30	2.00	0.0	-0.9
170	5.2	7.6	0.30	2.00	0.2	-0.9
180	4.8	7.0	0.30	1.50	0.3	-0.9
190	4.4	6.4	0.30	1.50	0.5	-0.9
200	4.0	5.7	0.30	1.50	0.6	-0.9
210	3.6	4.9	0.30	1.25	0.8	-0.8
220	3.2	3.9	0.30	1.00	1.0	-0.8

Table 1. Parameters of the final beam source model given as a function of the beam energy.

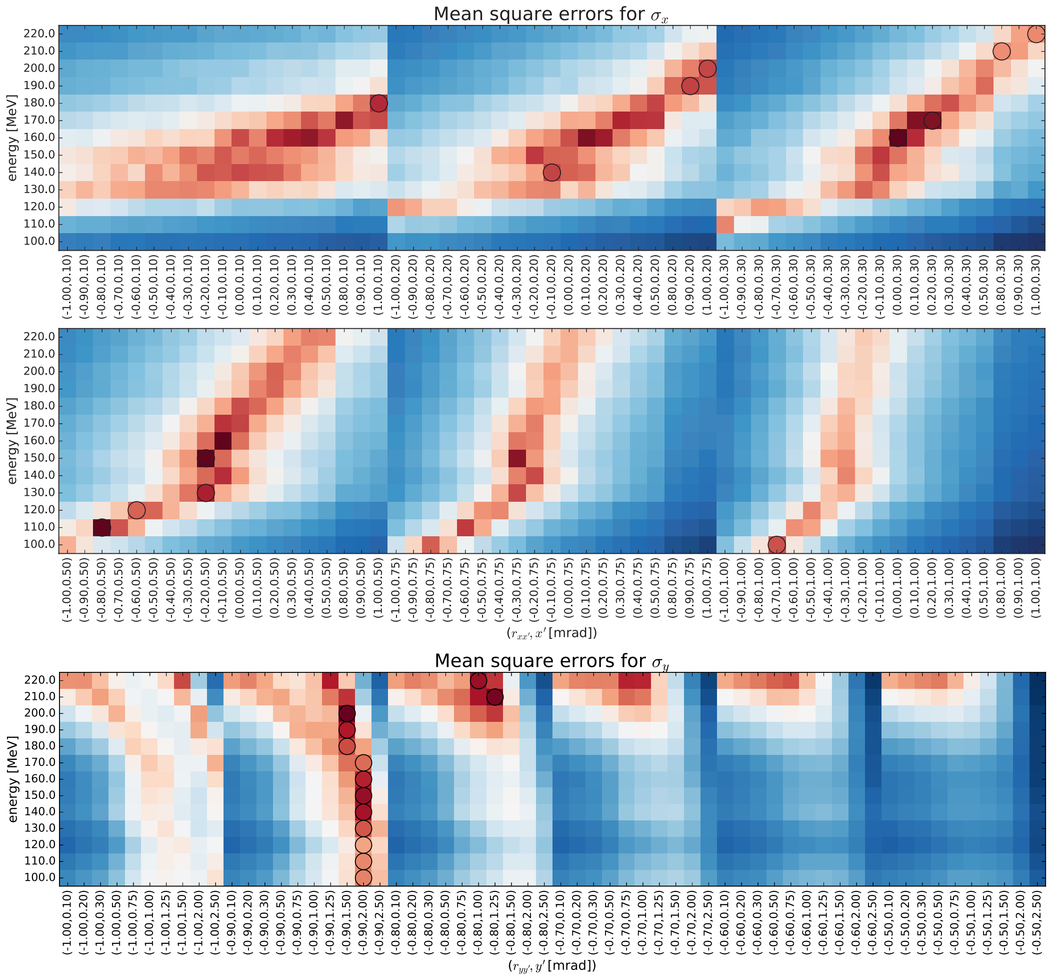

Figure 2. Fitting the beam source parameters $x', y', r_{xx'}$ and $r_{yy'}$: The coloured squares correspond to the logarithm of the mean squared error of the beam sizes at five positions around the isocentre. A red colour indicates a low error and good fit and the best fitting parameters are marked by circles.

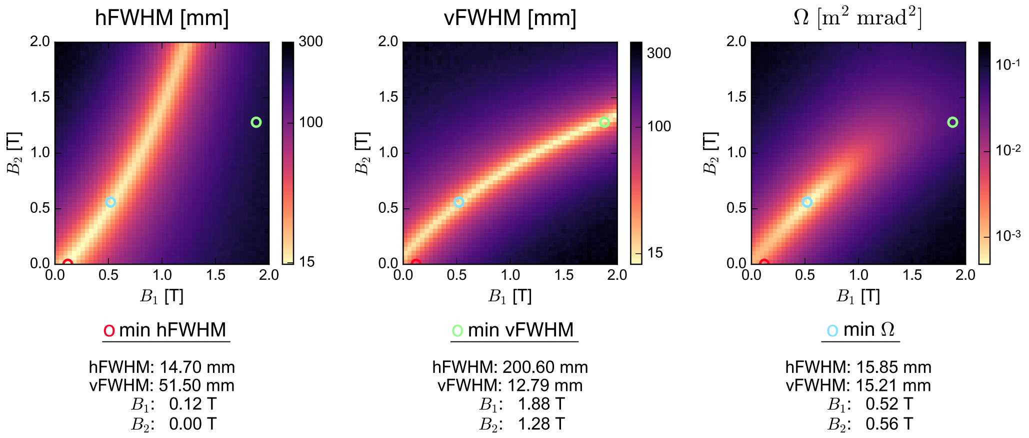

Figure 3. Beam size minimisation: hFWHM, vFWHM and Ω at the isocentre for the current geometry of the PBS nozzle as functions of B_1 and B_2 (field at the pole tips Q1 and Q2, respectively). Lighter colours signify smaller beam sizes and circles mark the minimum configurations. The corresponding minimum values are presented below each panel.
